# Alternative Oxidase (AOX) Senses Stress Levels to Coordinate Auxin-Induced Reprogramming From Seed Germination to Somatic Embryogenesis—A Role Relevant for Seed Vigor Prediction and Plant Robustness

**DOI:** 10.3389/fpls.2019.01134

**Published:** 2019-09-20

**Authors:** Gunasekaran Mohanapriya, Revuru Bharadwaj, Carlos Noceda, José Hélio Costa, Sarma Rajeev Kumar, Ramalingam Sathishkumar, Karine Leitão Lima Thiers, Elisete Santos Macedo, Sofia Silva, Paolo Annicchiarico, Steven P.C. Groot, Jan Kodde, Aprajita Kumari, Kapuganti Jagadis Gupta, Birgit Arnholdt-Schmitt

**Affiliations:** ^1^Plant Genetic Engineering Laboratory, Department of Biotechnology, Bharathiar University, Coimbatore, India; ^2^Functional Cell Reprogramming and Organism Plasticity (FunCROP), University of Évora, Évora, Portugal; ^3^Cell and Molecular Biology of Plants (BPOCEMP)/Industrial Biotechnology and Bioproducts, Department of Sciences of the Vidaydela Agriculture, University of the Armed Forces-ESPE, Milagro, Ecuador; ^4^Faculty of Engineering, State University of Milagro (UNEMI), Milagro, Ecuador; ^5^Functional Genomics and Bioinformatics Group, Department of Biochemistry and Molecular Biology, Federal University of Ceará, Fortaleza, Brazil; ^6^Council for Agricultural Research and Economics (CREA), Research Centre for Animal Production and Aquaculture, Lodi, Italy; ^7^Wageningen Plant Research, Wageningen University & Research, Wageningen, Netherlands; ^8^National Institute of Plant Genome Research, New Delhi, India; ^9^CERNAS-Research Center for Natural Resources, Environment and Society, Department of Environment, Escola Superior Agrária de Coimbra, Coimbra, Portugal

**Keywords:** environmental stress, developmental plasticity, metabolic biomarker, endophytes, seed technology, plant performance prediction

## Abstract

Somatic embryogenesis (SE) is the most striking and prominent example of plant plasticity upon severe stress. Inducing immature carrot seeds perform SE as substitute to germination by auxin treatment can be seen as switch between stress levels associated to morphophysiological plasticity. This experimental system is highly powerful to explore stress response factors that mediate the metabolic switch between cell and tissue identities. Developmental plasticity per se is an emerging trait for *in vitro* systems and crop improvement. It is supposed to underlie multi-stress tolerance. High plasticity can protect plants throughout life cycles against variable abiotic and biotic conditions. We provide proof of concepts for the existing hypothesis that alternative oxidase (AOX) can be relevant for developmental plasticity and be associated to yield stability. Our perspective on AOX as relevant coordinator of cell reprogramming is supported by real-time polymerase chain reaction (PCR) analyses and gross metabolism data from calorespirometry complemented by SHAM-inhibitor studies on primed, elevated partial pressure of oxygen (EPPO)–stressed, and endophyte-treated seeds. *In silico* studies on public experimental data from diverse species strengthen generality of our insights. Finally, we highlight ready-to-use concepts for plant selection and optimizing *in vivo* and *in vitro* propagation that do not require further details on molecular physiology and metabolism. This is demonstrated by applying our research & technology concepts to pea genotypes with differential yield performance in multilocation fields and chickpea types known for differential robustness in the field. By using these concepts and tools appropriately, also other marker candidates than AOX and complex genomics data can be efficiently validated for prebreeding and seed vigor prediction.

## Background

Environmental changes challenge plant plasticity at both individual and evolutionary levels. Moreover, environment can blur borders between hormone-related individual physiological characteristics and species-relevant, genetic traits by acting on meristems/stem cells (Cutri et al., 2013). Environmental changes are transmitted to plants *via* complex, diverse signaling. External signals are translated *via* secondary messengers to adaptive mild or severe molecular-physiological responses. Adaptive growth and developmental regulation associate to complex concentration- and spatiotemporal-dependent balances of networking hormones. Transcription and epigenetic factors are often rated as master regulators. They play critical roles during adaptation and link to genome-wide structural changes, such as copy number variability (CNV) and chromatin remodeling (Arnholdt-Schmitt, 2004; Gabur et al., 2019). This complex scenario connects to wide concerted gene networks, where DNA sequences form the structural primary basis for metabolism (Arnholdt-Schmitt, 2005a). Reactive oxygen species (ROS), redox-related pathways, and nitric oxide are known to integrate environment signaling *via* DNA break and repair mechanisms, hormone actions, and communication from cell to nucleus (retrograde communication) to adjust metabolism and physiology for individual genotype adaptation (anterograde communication). ROS induced ROS release’ (RIRR) is a process in which cellular compartments or organelles release ROS, which triggers production of ROS at other sites (Zandalinas and Mittler, 2018). RIRR is explored as crucial pathway to confront abiotic or biotic challenges. Crosstalk between ROS and autophagy linked to hormone signaling is associated to stress tolerance.

Mitochondria play a major role in managing stress response and cell network integration (Galluzzi et al., 2012). They adjust their structures, mobility, and activities upon external and internal stimuli to optimize growth and development *via* mitochondrial retrograde response signaling. Expression of transcription factor (TF) ANAC017 in complex hierarchy of 12 downstream TFs can lead to higher expression of genes related to mitochondrial stress and cell death/autophagy (Meng et al., 2019). Somatic embryogenesis (SE) is stress-inducible (Zavattieri et al., 2010). The balance between survival, embryo development, and programmed cell death (PCD) together with suspensor elimination seems critical for SE efficiency in angiosperms and gymnosperms (Smertenko and Bozhkov, 2014). Mitochondria play crucial role in manifesting two waves of PCDs during SE in conifers (in Arnholdt-Schmitt et al., 2016). They perceive signals from environment through adaptive membrane fluidity and transfer signals *via* respiration mediated by ROS for adaptive metabolic adjustment at cell, tissue, organ, and organism level. Metabolic profiling in young maize roots is promising for predicting heterosis in field (de Abreu E Lima et al., 2017). Calorespirometry is the only technology that enables linking temperature-dependent, metabolic adjustment based on respiration traits to growth performance (Hansen et al., 2005; Hansen et al., 2009; Scafaro et al., 2017). In a simple, rapid way, it allows exploring almost simultaneously rates of both heat emission and CO_2_ production (Hansen et al., 2005; Arnholdt-Schmitt, 2017). Calorespirometry data from *Prosopis cineraria* correlated closely with embryogenic tissue response. Therefore, calorespirometry was recommended as an early predictive tool for reprogramming events (Kim et al., 2006). In carrot, alternative oxidase 1 *AOX1* and *AOX2a* expression peaks during *de novo* growth induction, which coincided with a critical time point that can be used for biomass prediction using calorespirometry (Campos et al., 2016). In maize, salicylic acid–induced earlier seed germination could be sensed by increased heat rates (Moravcová et al., 2018). Calorespirometry enables studying in unicellular systems the effect of isolated *AOX* genes on growth performance linked to gross metabolism and cell density (Arnholdt-Schmitt and Patil, 2017). Recently, calorespirometry is being developed as a general tool for conventional and molecular plant/holobiont selection associated to plant robustness and germination efficiency (Arnholdt-Schmitt et al., 2015; Arnholdt-Schmitt et al., 2018).

## Carrot Seeds Provide a Powerful Experimental System to Study Cell Fate and Developmental Plasticity

Seed germination and induction of SE are adaptive responses to environmental changes. Germination of dry seeds occurs upon water imbibition in an oxygen-containing environment. This process, visible by radicle emergence, can be seen as environment-induced stress. Seeds recover from the dry and oxygen-low status and acclimate to water- and oxygen-enriching environment. On the other hand, SE is known as adaptive response to higher stress levels (Zavattieri et al., 2010; Fehér, 2014; Winkelmann, 2016). *In vitro* induction of SE is the most striking example of stress-inducible cell reprogramming due to totipotency acquisition (Frederico et al., 2009a). This response emerges when survival mechanisms are activated by extreme conditions. It was proposed that SE can be used as an experimental system to identify differential genotype behavior linked to stress tolerance (Frederico et al., 2009a; Afuape et al., 2013).

In early steps of zygotic and SE, auxin distribution plays a predominant role for polarity and pattern formation (Winkelmann, 2016; Robert et al., 2018; Figueiredo and Köhler, 2018). The effect of exogenous and endogenous auxin on growth and development depends on complex feedback networks associated to spatiotemporal biogenesis, transport, and gradients of endogenous auxin at the tissue and cell levels. Mitochondrial disturbance was unambiguously shown to be harmful for auxin signaling (Kerchev et al., 2014). Abscisic acid (ABA) and gibberellins are primary hormones that regulate seed dormancy and germination in a concentration-dependent manner (Miransari and Smith, 2014; Shu et al., 2016). However, ABA-mediated seed dormancy seems to be inevitably controlled through auxin-responsive factors (ARFs) besides micro RNAs (miRNAs) (Liu et al., 2013). Also, accumulation of endogenous auxins was found to be concomitant with germination initiation (Bialek et al., 1992). Germination can be promoted or inhibited through supplying auxin depending on its concentration, which is species-dependent (Garrard, 1954). In carrot seeds, zygotic embryos are still immature after desiccation (Homrichhausen et al., 2003). When carrot seeds are treated with high auxin from imbibition, germination is suppressed, and at the same time, embryogenic calli are induced that can later give rise to multiple propagules (Frederico et al., 2009a). Thus, the combined experimental system including germination and SE induction provides excellent opportunity to study changes between different levels of stress severity. This makes it especially useful to reveal relative importance of potentially relevant factors that interacts during stress adaptation.

## AOX—A Mere Stress Indicator/Alleviator or Rather an Efficient Coordinator of Adaptive Morphophysiological Plasticity?

Alternative oxidase is key enzyme in mitochondrial alternative respiration (AR), an additional cyanide-insensitive pathway for driving electrons to oxygen, thereby decreasing ROS production. This alleviates various stress conditions, including drought, low oxygen, and temperature. *AOX* gene family is nucleus encoded, composed of one to six members distributed in two subfamilies (*AOX1* and *AOX2*) (Costa et al., 2014; Costa et al., 2017). Vanlerberghe et al. (2009) postulated a “master role” for AOX through degree of homeostasis signaling. It can improve cell survival rates and seems to determine threshold for inducing PCD. This was shown in various organisms across kingdoms (Rogov et al., 2014; Fernandez Del-Saz et al., 2018). The role of AtAOX1a in maintaining cellular redox homeostasis, protecting cells against oxidative damage, improving the chance of survival and sustaining growth under oxidative stress in *Saccharomyces cerevisiae* was reported (Vishwakarma et al., 2016). Ivanova et al. (2014) highlighted reciprocal interaction of mitochondrial stress and auxin signaling for *AtAOX1a* regulation. It has been confirmed by many recent studies that AOX acts as a positive indicator for plant performance and the role of AOX in complex networks (Scheibe, 2018; Selinski et al., 2018; Wang et al., 2018). Positive effects are mediated mainly through *AOX* down-regulation with the help of transcriptional suppression and protein activity regulation that confers regulatory and metabolic flexibility (Fernandez Del-Saz et al., 2018; Selinski et al., 2018). AOX1 and AOX2 have differences in their conserved regions flanking CysI (conserved cysteine residue) (Costa et al., 2009a; Costa et al., 2014). They are differentially regulated by tricarboxylic acid cycle metabolites related to different structures and functionalities (Albury et al., 2009; Elliott et al., 2014; Selinski et al., 2017). However, Anthony Moore stressed that AOX activity conferred by all gene family members together is important (personal communication), since the mechanism of enzymatic activity is the same, irrelevant of *AOX* gene and organism (Elliott et al., 2014). Coregulation versus differential regulation of *DcAOX1* and *DcAOX2a* in carrot was connected to earlier or later stages in embryo development (Frederico et al., 2009a). Co- upregulated transcription of both genes was observed during *de novo* growth induction from quiescent root phloem tissue (Campos et al., 2016).

In 2006, the hypothesis was raised that *AOX* can serve as a functional marker for efficient cell reprogramming under stress (Arnholdt-Schmitt et al., 2006; Clifton et al., 2006; Arnholdt-Schmitt, 2015), and a symposium and special issue (Physiol Plantarum, 2009) were exclusively dedicated to its role in yield stability. The role of AOX for target cell or tissue reprogramming and plant plasticity was studied in diverse *in vitro* and *in vivo* systems, such as in olive rooting (Santos Macedo et al., 2009; Hedayati et al., 2015; Velada et al., 2018), in carrot SE (Frederico et al., 2009a; Arnholdt-Schmitt et al., 2016), carrot primary cultures (Campos et al., 2009; Campos et al., 2016), and germination of *Hypericum perforatum* (Velada et al., 2016). *AOX* gene diversity is characterized extensively (Costa et al., 2009b; Polidoros et al., 2009; Cardoso et al., 2015; Nobre et al., 2016; Nogales et al., 2016a), and the importance of ecotyping (Costa and Svensson, 2015), epigenetics (Noceda et al., 2015; Costa and Arnholdt-Schmitt, 2017), and new software based on artificial intelligence (Quaresma et al., 2015) for functional marker development was highlighted. Considering the plant–endophyte interaction for functional genomics was stressed in general (Arnholdt-Schmitt et al., 2014; Nogales et al., 2016b), and involvement of AOX from both partners in symbiotic systems is indicated (Campos et al., 2015; Mercy et al., 2017).

During soybean germination, Yentur and Leopold (1976) observed transition from alternative to normal respiration at 4 to 8 hours after seed imbibition (HAI). AR was linked to germination initiation, seedling growth, and chlorophyll synthesis. Similar results were reported in other species (Esashi et al., 1979). In cocklebur seeds, AR increased by 5 to 7 HAI and contributed heavily to germination at its earliest stage. By applying AOX-inhibitor salicylhydroxamic acid (SHAM) to seeds, Esashi et al. (1981) showed that the most sensitive phase to SHAM ranged from 6 to 12 HAI. These authors also observed that seeds with higher germination potential showed higher capacities of AR. In *Orobanche*, a parasitic plant, “clear-cut effect” germination through AOX inhibition corresponds to changes in oxygen consumption and had functional implication for controlling germination and pathogenicity (Nun et al., 2003). In early stages, germination was sensitive to lower O_2_-tension, indicating relevance of AR, since AOX has lower affinity to O_2_ than cytochrome oxidase (Bonner, 1973, cited in Yentur and Leopold, 1976). *Avena fatua* showed maximal germination efficiency when seeds were treated by cyanide or azide during 4 to 16 and 4 to 12 HAI respectively. While stimulation of respiration by azide appeared to be SHAM sensitive, once induced, both azide- and cyanide-stimulated respirations were insensitive to SHAM (Tilsner and Upadhyaya, 1987). In dry and mature seeds of *Arabidopsis thaliana*, and during early germination, Saisho et al. (2001) and Clifton et al. (2006) found high expression of *AOX2*, with a rapid decrease of transcripts after 12 HAI. However, AR capacity seemed dependent on both family members, *AOX2* and *AOX1a* (Saisho et al., 2001). A critical role of ROS for germination was indicated by positive effects of H_2_O_2_ and adverse response to AOX inhibition; the importance of exogenous H_2_O_2_ in germination was also confirmed in other plant species (Barba-Espín et al., 2012). Auxins can induce oxidative stress and substitute H_2_O_2_ treatment. In olive, auxin-dependent *de novo* root induction was linked to H_2_O_2_ (Santos Macedo et al., 2009, and references herein). An inhibitory effect of SHAM on olive rooting was observed in micro-shoots and semi-hardwood cuttings (Santos Macedo et al., 2012; Porfirio et al., 2016). SHAM inhibition appeared to be specific to rooting and did not interfere with preceding callus formation (Santos Macedo et al., 2012). Promising association between rooting ability and *OeAOX2* gene polymorphism in olive cuttings was first suggested by Santos Macedo et al. (2009) and later confirmed in genetic studies by Hedayati et al. (2015). Recently, Velada et al. (2018) associated *OeAOX1a* and *OeAOX1d* transcript accumulation to induction, initiation, and expression phase of rooting. Both genes were coregulated and dramatically increased in early stages without visible root induction with a maximum peak of transcript accumulation at 8 HAI and rapid decrease from 1 DAIs (days after imbibition). At 4 DAIs, a second smaller peak was observed corresponding to initiation of first meristemoids and morphogenetic zones. A third increment occurred for both genes around 10 to 14 DAIs, which corresponds to callus formation from 12 to 14 DAIs before root emergence starts at around 22 DAIs. At the earliest increments, *OeAOX1a* was more pronounced than *AOX1d*, and higher transcript levels were observed at 14 DAIs when cell division activity increased. It remains to be seen whether differences in transcript accumulation might be related to distinct auxin responsive elements observed within both promoter regions.

In SE, less information is available on AR, but a role of AOX was proposed by Arnholdt-Schmitt et al. (2006; 2016). In *Abies alba*, AOX was found to act during SE induction *via* ROS capturing as an anti-apoptotic factor (Petrussa et al., 2009). Frederico et al. (2009a) reported early up-regulation of *AOX* transcripts during initiation of carrot somatic embryo development upon auxin depletion. Transcript level increase was consistent with a 2.5-fold up-regulated *AOX1* and 1.5-fold down-regulated *AOX2a*. During late embryo development, differential regulation was observed, and it was reversibly suppressed by SHAM supply and depletion. This research was aimed to identify promising marker candidates for assisting SE in recalcitrant species such as conifers, finally promising functional marker candidates could be identified from *DcAOX2b* (Frederico et al., 2009b; Frederico, 2017).

**Figure 1:**
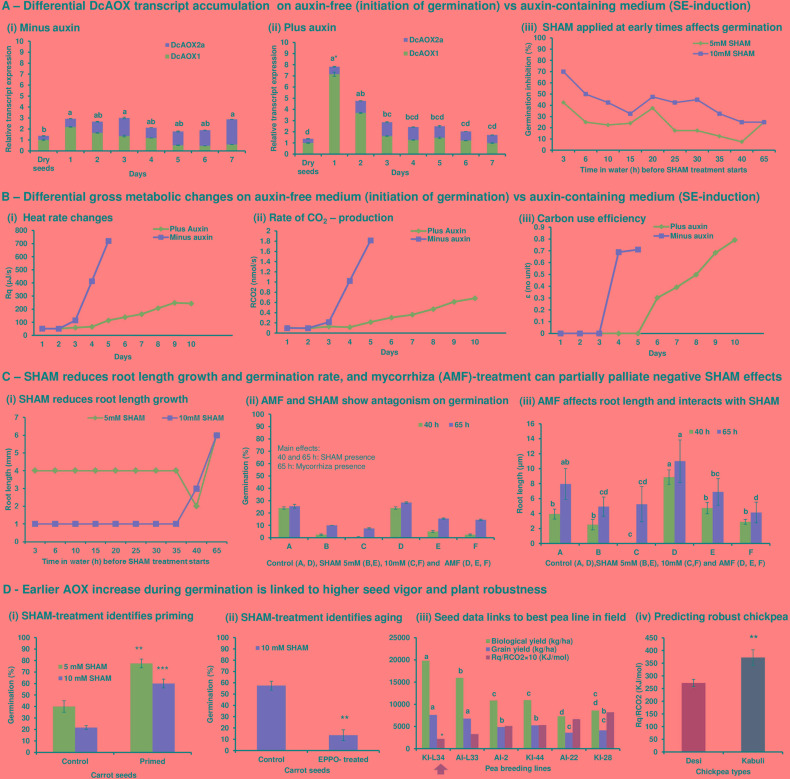
**(A)** Differential DcAOX transcript accumulation on auxin-free (initiation of germination) versus auxin-containing medium (SE induction). At day 1 of carrot seed cultivation with and without auxin, a significant increase in AOX transcript accumulation was observed **(A**i, ii**)**. This was mainly due to AOX1 (**Figure S1**) and confirms its role as stress indicator as shown in many other systems (see references in text). However, when SE was induced, this peak was strikingly higher, which indicates higher stress. In the following days, the level of AOX transcripts remained stable in the absence of auxin, since decreasing levels of AOX1 were compensated by slightly variable, but increasing levels of AOX2a. In this equilibrated situation, growth of seed embryos were initiated, which was visible by root emergence. AOX2 transcript levels increased to significantly higher values at day 7 when seedling growth was established. On the contrary, when SE was induced, slower stress recovery was indicated; the high peak of AOX transcripts was rapidly going down due to significantly decreasing amounts of AOX1 transcripts. However, AOX2a transcripts remained at stable level. From day 4, the overall transcripts was stabilized at a low level. In auxin-containing medium that mediated SE induction, no increase in AOX2a could be observed during the experimental time period **(A**ii**)**. Sequential SHAM inhibition during the initiation of germination confirmed that most critical events for germination happen from 3 to 15 HAI **(A**iii**)**. **(B)** Differential gross metabolic changes on auxin-free medium (initiation of germination) versus auxin-containing medium (SE-induction). From day 2 of carrot seed imbibition, i.e. 1 day after AOX stress signaling was observed, metabolic heat emission rate **(B**i**)** and rate of CO2 production **(B**ii**)** increased. At that time, we started to observe emergence of radicles. From day 3, carbon was efficiently translated into growth indicated by an increased value for carbon use efficiency (CUE) **(B**iii**)**. When SE was induced by auxin at imbibition, metabolic heat emission rate was transiently suppressed, and a slighter increase in heat rate and RCO2 started only after day 4. Nevertheless, this increase did not reach the same level as observed already at day 4 during germination and remained still low until end of experiment at day 10 after imbibition. While during germination CUE increased rapidly to 0.7, a value that indicates cell division growth (Hansen et al., 2005), during SE this value was reached only from day 9, which can thus be supposed to indicate initiation of embryonic callus formation. **(C)** SHAM reduces root length growth and germination rate and mycorrhiza (AMF) treatment can partially palliate negative SHAM effects. Sequential SHAM treatment during the first 35 HAI changed the mean length of emerging roots in comparison to control seeds (only in water until 65 HAI) in a concentration-dependent manner when observed at 65 HAI **(C**i**)**. However, no differential effects were observed when treatment start varied from 3 to 35 HAI. When SHAM was supplied only at 40 HAI, root length could no longer be differentially affected by inhibitor concentration. Influence of SHAM and AMF on the germination rate and root length was represented in graphs **(C**ii, iii**)**. AMF-inoculated seeds increased germination efficiency by affecting root length growth rather than by affecting early initiation of germination. AMF interacted with SHAM treatment on root growth and could partly compensate SHAM-reduced germination rate. Additionally, endophytes available in the seeds blocked the positive effect of added AMF on root growth (visible already at 40 HAI) and affected % of germination only when observed at 65 HAI (shown in **Figure S2**). Differences in root length between treatments for each time are stated with different letters (α = 0.05). **(D)** Earlier AOX increase during germination is linked to higher seed vigor and plant robustness. As proof-of-concept trials, control and primed, coated commercial F1 carrot seeds (cv. Nerac 2) **(D**i**)**, control, and elevated partial pressure of oxygen (EPPO) stress–treated carrot seeds (cv. Nantaise 2/Milan) **(D**ii**)**, Pea seeds from breeding lines with top-ranking, mid-ranking and bottom ranking biological and grain yielding ability over three test environments (Annicchiarico et al., 2019) were compared for 18 each of two RIL populations **(D**iii**)** and two chickpea types known for differential yield performance and multistress tolerance in field were compared **(D**iv**)**. **(D**i**)** Primed, coated seeds show higher germination efficiency and have improved seedling vigor in field (seeds and information provided by BejoSamen). These seeds show increased earlier metabolic heat emission rate and CO2 production (data not shown). When treated with SHAM from 10 HAI, germination efficiency in primed, coated seeds could not be reduced as strongly as the control. This is congruent with our expectation that AOX signaling is critically relevant for germination efficiency. From the described results **(Panels A**–**C)**, primed seeds could be expected to demonstrate an earlier stress-related AOX peak than control seeds and a more efficient stress recovery indicated by rapidly lowered AOX1 transcript levels. Thus, primed seeds could supposedly be less sensitive to early SHAM inhibition at 10 HAI during germination. In fact, this could be shown homogeneously across all three repetitions by using 3× bulked samples of 40 seeds. Further, heat rate increase of primed seeds could completely be suppressed when SHAM was applied at 2 HAI, while this did not happen in control seeds where heat rate increase was only postponed (data not shown). **(D**ii**)** EPPO-stressed seeds induce aging and showed already 2 weeks after having treated the dry seeds, a significantly reduced speed of germination at T50 (data not shown). In agreement with the expectation that higher vigor control seeds are at the start of SHAM treatment at 10 HAI, which are already less sensitive to the AOX inhibitor, EPPO-stressed seeds display lower germination rates homogeneously in all three repetitions. **(D**iii**)** Pea breeding lines that were grown by a breeder in three locations demonstrated significant differences in yield performance. Only the best breeding line KI-L34 was selected by the breeder for cultivar registration based on complex field data. By applying calorespirometry at 10 HAI at a constant temperature (25°C) and using oxycaloric equivalent (Rq/RCO2) values, the breeding lines could be ranked a posteriori with an inverse relationship to yield data. The breeder-selected line for registration was in fact the only one, significantly different from all others. Thus, applying our method would provide a highly innovative, predictive biomarker for early plant selection on yield ability. **(D**iv**)** Early chickpea plant vigor is critical for plant productivity under terminal drought conditions (Sivasakthi et al., 2017). From the two principle chickpea types, Desi and Kabuli, it is known from vast field experience that Desi is clearly superior in terms of multistress tolerance and yield performance (Purushothaman et al., 2014). By applying our approach, we can discriminate both types and predict a posteriori the known better yield stability of Desi by a lower oxycaloric equivalent (Rq/RCO2) value due to differential carbon use at 10 HAI.

In **Figure 1**, the advanced rationale of our perspective on the coordinative role of AOX in stress management and its practical impact for plant holobiont breeding and applied functional hologenomics (Nogales et al., 2016b) are explained. It starts from original research (A–C) until proof-of-concept validation (D) (methods are provided in Supplementary file). In conclusion, we found that (a) the level of early *AOX* transcript stress signaling can be relevant and associated to subsequent changes in metabolism and morpho-physiology; (b) when strong morphological reprogramming is induced (by adding highly concentrated, stressful auxin that induces SE) versus expression of already induced morpho-physiology (seed imbibition in auxin-free medium that initiates germination), an early higher *AOX* transcript peak appears (**Supplementary Figure S1**); (c) higher early AOX stress signaling is linked to slower AOX stress signaling recovery associated to slower global metabolic adjustment and increase in carbon use efficiency (CUE); (d) very first hours of seed germination are relevant for both, individual development (germination efficiency, seed vigor) and identification of differences in seed vigor due to genetics or seed treatment; (e) differences in seed vigor and plant field performance can be predicted from early hours after seed imbibition by SHAM-inhibition trials and global metabolism data based on respiration traits (oxycaloric equivalent); (f) arbuscular mycorrhizal fungi (AMF) inoculation interfered with germination efficiency affecting root length growth rather than by initiating germination; (g) AMF inoculation interacts with SHAM treatment on root growth and palliates effects of the inhibitor on germination efficiency (**Supplementary Figure S2**); (h) AMF inoculation effects can be modified by endophytes available in seeds.

To validate generality of our insights, we searched for *in silico* data. We reviewed public transcriptome data on germination of *Arabidopsis* and soybean and on auxin-induced SE from *Arabidopsis* wild type (Col-0) and nonembryogenic mutant clf/swn (**Table 1**). Taken all findings together, higher initial expression of *AOX1a* appears to be essential for efficient root emergence, while higher expression of *AOX2* (a/b/c type) is required for further development. Related to SE, in wild type (Col-0), *AtAOX1* transcript increase was important to overcome epigenetic SE barriers. It was potentiated through injury, while *AOX2* expression remained stable. In clf/swn, *AOX1* did not increase, and *AOX2* was barely detected. Appropriate *in silico* studies on plant–endophyte interaction were not available from germination trials and SE. However, endophytes modulate *AOX* transcripts in a species-, stress-, and development-dependent manner (**Supplementary Table S1**). It is confirmed that endophytes interact specifically with stress-related *AOX* gene family members. In *Arabidopsis*, salt stress increased *AOX1a* expression, while *Enterobacter* species reduced its expression. However, when salt stress was applied together with endophytes, mRNA levels of *AOX1a* were maintained low (de Zélicourt et al., 2018) (**Table S1**).

**Table 1:** Expression of *AOX* genes during germination (*Arabidopsis* and soybean) and somatic embryogenesis (*Arabidopsis*) using RNA-seq data.

Species		Bioproject	Tissue/Genotype	Sample	Replicate number	AOX gene expression (RPKM)						References
										AOX2		
						AOX1a	AOX1b	AOX1c	AOX1d	AOX2a	AOX2d	
**Germination**	*A. thaliana*	PRJNA369750	Dry seed	Seed- 0h	3	1.25^a^	0.008^a^	0.009^a^	0.15^a^	11.8^a^		Narsai et al., 2017
			Germinating seed	Soaked in light exposure – 1 h	3	1.6^a^	0^a^	0.14^ab^	0.011^b^	6.45^b^		
				Soaked in light exposure – 6 h	3	1.2^a^	0^a^	0.3^ab^	0.006^c^	6^b^		
				Soaked in light exposure – 12 h	3	0.5^a^	0^a^	0.31^ab^	0.002^c^	1.65^c^		
				Soaked in light exposure – 24 h	3	5.8^b^	0.003^a^	0.96^c^	0.006^c^	1.21^c^		
			Seedling	Soaked in light exposure – 48 h	3	9.6^c^	0.003^a^	0.25^a^	0^c^	0.1^c^		
		PRJNA415950	Col-0 control	Dry seed	3	0.06^a^	0^a^	0.02^a^	0.12^a^	28.9^a^		Jin et al., 2018
				Germinating – 48 h	3	0.46^b^	0^a^	0.1^b^	0.016^b^	3.1^b^		
			*csn5b-1* (high germination rate)	Dry seed	3	0.1^a^	0^a^	0.008^a^	0.1^a^	19.9^c^		
				Germinating – 48 h	3	0.26^b^	0^a^	0.13^b^	0.007^b^	2.2^b^		
			*csn5a-1* (retarded seed germination)	Dry seed	3	0.56^b^	0^a^	0.04^ab^	0.37^c^	20.3^c^		
				Germinating – 48 h	3	0.02^a^	0^a^	0.06^ab^	0^b^	0.36^d^		
			*csn1-10* (stronger seed dormancy)	Dry seed	3	0.34^b^	0^a^	0.008^a^	0.21^c^	26.4^a^		
				Germinating – 48 h	3	0.18^b^	0^a^	0.008^a^	0^b^	2.3^b^		
	*G. max*	PRJNA326110	Dry seed embryo	Dry seed- 0 h	2	3.8^a^	–	–	–	36.9^a^	44.7^a^	Bellieny-Rabelo et al., 2016
			Soaked embryo seed	Soaked- 3 h	2	6.2^b^	–	–	–	35.3^a^	47.9^a^	
				Soaked- 6 h	2	5.3^b^	–	–	–	24.8^b^	30.9^b^	
				Soaked- 12 h	2	6^b^	–	–	–	11.9^c^	16.9^c^	
				Soaked- 24 h	2	6.1^b^	–	–	–	5.4^c^	20.12^c^	
		PRJNA325298	TW-1 (very low rate of seed field emergence)	Dry seed – 0 h	3	3.25^a^	–	–	–	17.1^a^	32.2^a^	Yuan et al., 2017
				Soaked- 12 h	3	3.9^a^	–	–	–	13.7^a^	35.5^a^	
				1st emerging root	3	1.75^b^	–	–	–	6.6^b^	13.5^b^	
			TW-1-M (higher rate of seed field emergence)	Dry seed- 0 h	3	3.1^a^	–	–	–	9.6^a^	29.9^a^	
				Soaked- 12 h	3	2.7^a^	–	–	–	4.19^c^	19.8^a^	
				1st emerging root	3	4.7^c^	–	–	–	2.98^c^	25.6^a^	
**Somatic embryogenesis**	*A. thaliana*	PRJNA320769	Col-0 reference	Control- 0 h	3	7^a^	0.01^a^	0.31^a^	0.06^a^	0.01^a^		Mozgová et al., 2017
				Control- 55 h	3	7.06^a^	0.01^a^	0.29^a^	0.6^a^	0.03^a^		
				Auxin induction medium- 55 h	3	17.6^b^	5.8^b^	3.11^b^	9.4^b^	0.3^a^		
				Injury induction medium- 55 h	3	9.4^a^	0.03^a^	0.4^a^	1^a^	1.1^a^		
				Injury + auxin induction medium – 55 h	3	31.5^c^	8.95^c^	5.6^c^	13.2^c^	0.06^a^		
			Clf/swn (mutant without somatic embryogenesis epigenetic barrier)	Control- 0 h	3	2.85^a^	0^a^	0.02^a^	0^a^	6.5^b^		
				Control- 55 h	3	3.3^a^	0^a^	0^a^	0.05^a^	5.3^b^		
				Aux in induction medium- 55 h	3	2.7^a^	0^a^	0.01^a^	0^a^	5.3^b^		
				Injury induction medium- 55 h	3	5.2^a^	0.03^a^	0.02^a^	0.05^a^	2.1^c^		
				Injury + auxin induction medium – 55 h	3	9.46^a^	0.02^a^	0^a^	0.02^a^	3.3^c^		

The AOX expression was performed according to Saraiva et al. (2016). Gene expression data were statistically analyzed using the Prism tool (GraphPad Prism), through the variance analysis by analysis of variance with the Tukey test parameters. Lower case letters represent the comparison of treatment conditions. Same letters indicate that no statistical difference between time/treatments was observed.

## Outlook

Biologists aim to understand biological and ecological complexity. However, for economy, it is important that biological systems work properly as expected. This contrasting view from application is the reason why top-down approaches are required for crop improvement rather than bottom-up strategies starting with complexity (Arnholdt-Schmitt, 2005a). First, species-specific target tissues/cells need to be identified that associate to outlined traits, such as yield stability; for example, adaptive growth and development responses depend on stem cells, and molecular characterization needs to start at that cell level. This insight is widely agreed within the community working with AOX (Arnholdt-Schmitt, 2005b; Nogales et al., 2015; Ragonezi and Arnholdt-Schmitt, 2017, Fernandez Del-Saz et al., 2018; Selinski et al., 2018). For breeding purposes, early selection at seed level is highly advantageous. Individual genetic information is physically concentrated due to high percentages of stem cells. Thus, it is easily accessible for characterization without requiring necessarily tissue or cell isolation. Moreover, during seed imbibition, the embryo is still at an early point of epigenetic programming and provoked for the first time to show its basic genetic capacity for stress management. These might be the reasons why successful prediction of later plant responses can already be done from germinating seeds.

Biological systems are unique and have their individual internal complexity that drives adaptive responses. Nevertheless, breeding and agricultural management can improve the probability that biological systems will function as expected. This strategy was successful over centuries. However, biological, ecological, and socio-economical systems underlie permanent, mutually dependent changes and require continuous adaptation. Plants with high general plasticity or robustness provide a better chance to sustain changing complex conditions and to enable the highest economic output.

Until the 1970s, it was believed that individual enzymes might play a key role in crop improvement for stable yields. However, this view changed (see in Arnholdt-Schmitt, 2005a) along with growing knowledge on biological complexity, the crucial role of quantitative genetics, and the importance of (not only social, but also) biological and genome-wide networks (systems biology). This is also the reason why a master role of AOX has been recently questioned (Scheibe, 2018; Wang et al., 2018). It is not easily accepted that one enzyme or pathway might play a critical, unique role for determining general fitness and productivity of plants. This drastic view is related to believing more in networks instead of individual components. However, the presented new fundamental insights applied to seeds with differential vigor strengthen our perspective that the small enzyme “clan” of AOX can play a key coordinative role for predicting seed and plant performance. Nevertheless, this view does not exclude that other types of marker candidates may also be successful. SHAM inhibits AOX, but it can affect also other enzymes and pathways. In contrary to other AOX inhibitors, such as propyl gallate, SHAM distinguishes *AOX* gene variants (Berthold, 1998), which might make this inhibitor especially interesting for genotype discrimination. Therefore, studying complex effects of SHAM on initiating germination versus SE in the proposed experimental carrot system might help identifying further molecular or metabolic candidates for genotyping. However, our extended *in silico* search (not shown) for candidates from auxin metabolism (Porfirio et al., 2016), urease, and others strengthened the master role of AOX. Besides, we got hints for a crucial role of growth- and development-related, genome-wide CNV for the most important repetitive elements in plants. Thus, it remains to be explored whether AOX functionality connects also to adaptive growth determination *via* global genome regulation as indicated by Arnholdt-Schmitt (1993, 1995, and 2004).

## Data Availability

All datasets generated for this study are included in the manuscript and the **Supplementary Files**.

## Author Contributions

GM performed *AOX* transcript analyses, calorespirometry, and SHAM studies demonstrated in **Figures 1A, B and C**i as part of her PhD studies. RB measured endophyte effects (**Figures 1C**ii + iii). BA-S analyzed primed seeds (**Figure 1D**i), and EM elevated partial pressure of oxygen (EPPO)–treated seeds (**Figure 1D**ii). EPPO treatment was performed by SG and JK. SS together with EM analyzed pea genotypes; pea lines had been bred and produced in multi-location field trials by PA; JC and KL performed all *in silico* studies (**Table 1** and **S1**). CN performed statistics analyses on all carrot trials and together with JC was strongly involved in interpreting all data; RS supervised together with SRK the laboratory work of GM and RB. AK and JGK proposed and integrated chickpea studies, and AK performed together with EM calorespirometry analyses. All were involved in manuscript writing and data interpretation. SRK managed final organization of the manuscript for submission; all coauthors approved the final manuscript. BA-S designed and coordinated the overall project.

## Conflict of Interest Statement

The authors declare that the research was conducted in the absence of any commercial or financial relationships that could be construed as a potential conflict of interest.
