# Supplementary material for: Alternative Oxidase (AOX) Senses Stress Levels to Coordinate Auxin-Induced Reprogramming From Seed Germination to Somatic Embryogenesis—A Role Relevant for Seed Vigor Prediction and Plant Robustness
Source: Front Plant Sci. 2019 Sep 20;10:1134. doi: 10.3389/fpls.2019.01134 (PMC6776121; doi:10.3389/fpls.2019.01134)
Supplement: Supplementary file 1 [file DataSheet_1.docx]

**Alternative Oxidase (AOX) Senses Stress Levels to Coordinate Auxin-Induced Reprogramming from Seed Germination to Somatic Embryogenesis – A Role Relevant for Seed Vigor Prediction and Plant Robustness**

Gunasekaran Mohanapriya^1,2^, Revuru Bharadwaj^1,2^, Carlos Noceda^2,3,4^, José Hélio Costa^2,5^, Sarma Rajeev Kumar^2^, Ramalingam Sathishkumar^1,2*^, Karine Leitão Lima Thiers ^5^, Elisete Santos Macedo^2^, Sofia Silva^2^, Paolo Annicchiarico^6^, Steven P.C. Groot^7^, Jan Kodde^7^, Aprajita Kumari^8^, Kapuganti Jagadis Gupta^2,8^ and Birgit Arnholdt-Schmitt^2,5,9*^

1. Plant Genetic Engineering Laboratory, Department of Biotechnology, Bharathiar University, Coimbatore, Tamil Nadu, India
2. Functional Cell Reprogramming and Organism Plasticity (*FunCROP*), University of Évora, Évora, Portugal
3. Cell and Molecular Biology of Plants (BPOCEMP) / Industrial BiotechnologyandBioproducts, Department of Sciences of the Vidaydela Agriculture, University of the Armed Forces-ESPE, Ecuador.
4. Faculty of Engineering, State University of Milagro (UNEMI), Milagro, Ecuador.
5. Functional Genomics and Bioinformatics Group, Department of Biochemistry and Molecular Biology, Federal University of Ceará, Fortaleza, Brazil
6. Council for Agricultural Research and Economics (CREA), Research Centre for Animal Production and Aquaculture, Lodi, Italy
7. Wageningen Plant Research, Wageningen University & Research, Wageningen, The Netherlands
8. National Institute of Plant Genome Research, Aruna Asaf Ali Marg, New Delhi, India
9. CERNAS-Research Center for Natural Resources, Environment and Society, Department of Environment, Escola Superior Agrária de Coimbra, Coimbra, Portugal

# *Correspondence:

**Birgit Arnholdt-Schmitt**

University of Évora

Apart.94,7002-554Évora,

Portugal. Email: [eu_chair@uevora.pt](mailto:eu_chair@uevora.pt)

***Co Correspondence:**

Dr. R. Sathishkumar

Plant Genetic Engineering laboratory

Department of Biotechnology

Bharathiar University

Coimbatore 641 046

Tamil Nadu, India.

Email: [rsathish@buc.edu.in](mailto:rsathish@buc.edu.in)

**Supplementary Material and Methods (Figure and legend in text)**

**Germination and somatic embryogenesis (SE) of *Daucus carota L.* seeds on auxin-free and auxin containing medium**

*D. carota* seeds were surface sterilized with 75% of ethanol for 1 minute, 4% of sodium hypo chloride solution for 20 minutes and washed 3 times with sterile water. 25 seeds per plate were inoculated in auxin-free B_5_ solid medium (modified Gamborg et al., 1968) for germination and supplemented with auxin (0.5 mg/L 2,4-D) for SE induction as reported previously (Frederico et al., 2009). Plates were incubated at 25˚C at 16 h photoperiod shift. A total number of 500 seeds were inoculated for experiments and 40 Seeds were harvested every day until 5-10 days for analysis of *DcAOX* transcripts (cv. Kuroda, performed in India) and calorespirometry (cv. Rotin, performed in Portugal). Both varieties displayed similar behavior in germination with root emergence starting from 24 to 40 hours. Cv. Rotin embryogenic calli production one day earlier.

**Relative transcript accumulation of *AOX* genes by RT-qPCR**

**Fig. A (i, ii):** Total RNA was extracted from 40 seeds of cv. Kuroda by using TRIzol reagent (Invitrogen, CA, USA). DNase digestion was performed to remove DNA using DNaseI (Invitrogen, CA, USA) as described by the manufacturer. Total RNA was quantified using NanoDrop^TM^2000 Spectrophotometers (Thermo Scientific, Wilmington, DE, USA). First-strand cDNA was synthesized using 2µg of total RNA with First Stand cDNA Synthesis Kit (Thermo Scientific, USA) following manufacturer’s instructions.

For RT-qPCR, the reaction mixture of 10µL consisted of 25ng cDNA, 5 µL SYBR^®^ Premix Ex Taq^TM^II with ROX reference dye II (Takara Bio Co. Ltd. Dalian, China) and 5mM gene-specific primers, which have been previously used (Frederico et al., 2009; Campos et al., 2016). Reaction conditions for RT-qPCR were as follows: 94°C for 10 min, followed by 40 cycles of 94°C for 15 s, 60°C for 15 s and 72°C for 15 s. The Ct values were obtained from samples using Applied Biosystems 7500 Real-Time PCR systems (Applied Biosystems, Santa Clara, CA). Elongation factor 1α and β-actin were used as endogenous genes for normalization. Three sets of technical replicates were performed for each gene and results provided are the average of three replicates. GeNorm algorithm was used to determine the expression stability of housekeeping candidate genes and calculate a reliable normalization factor. The ratio between the row relative quantities and the corresponding normalization factor was calculated for each sample (Velada et al., 2016).

Statistics on *AOX* expression during germination and SE in carrot seeds were performed with InfoStat version 2018. For relative accumulation of *AOX* transcripts, two-way (treatment x time) ANOVA followed by Tukey test was performed: differences between treatments are stated with different letters and differences between treatments inside a time with asterisks (α = 0.05). To keep ANOVA pre-requisites the variables were transformed: square root neperian logarithm for *AOX1*, square root for *AOX2a* and neperian logarithm for accumulative values.

**Calorespirometry studies:**

**Fig. B (i), (ii) and (iii)**: Calorespirometry was performed in isothermal mode in model 4100 Multi-Cell Differential Scanning Calorimeter (TA Instruments, USA). Bulked samples of 40 seeds (cv. Rotin) were run in parallel in two repetitions (basic protocols in Hansen et al., 2005; Arnholdt-Schmitt, 2017).

**Salicylhydroxamic acid (SHAM)-mediated seed germination**

**Fig. A (iii) and C (i):** 40 seeds of *D. carota* L cv. Kuroda were inoculated in 90 mm petri dishes containing moistened filter paper. For inhibition studies, 5 or 10 mM SHAM (Sigma Aldrich, USA) was dissolved in 1 mL of DMSO and further diluted with sterile water to prepare desired concentrations. DMSO with water was used as control. In 'pulse’ treatments, seeds were imbibed in dishes with sterile water for 3, 6, 10, 15, 20, 25,30, 35, 40, 65 h and then transferred to dishes containing 5-mM and 10-mM SHAM. 40 seeds were inoculated in each petri dish and two petri dishes were maintained for each treatment. All plates were incubated at 25˚C in dark for 24 h till emergence of radicle and then plates were transferred to light. Germination and root length effect was recorded at 65 h after incubation in 16/8 photoperiod (Botha et al., 1986; Basra et al., 1989).

**Mycorrhiza-mediated seed germination and interaction with SHAM-treatment**

**Fig. C (ii), (iii); Figure S2 (i), (ii):** Two different sets of seeds were used for this study (i) Endophyte free seeds, (ii) Surface sterilized seeds. To generate endophyte free seeds, *D. carota* cv. Kuroda seeds were treated with 0.5% carbendazim and 0.5% streptomycin sulphate solution. Seeds were soaked in this solution for 6 h under constant shaking at 120 rpm. Later, seeds were washed twice with sterile deionized water and dried for 15 min under laminar air flow. To check the effectiveness of treatment, seeds were cut into small pieces and inoculated on potato dextrose agar (Hi Media Laboratories Pvt Ltd) kept at 28^0^C for 7 days. Three replicates were maintained for surface sterilization check. To generate surface sterilized seeds, seeds were surface sterilized using 70% alcohol for 3-5 min and subsequently with 4% sodium hypochlorite for 15 min. To remove traces of sodium hypochlorite, seeds were washed thrice with sterile distilled water.

Mycorrhiza spore suspension was prepared by adding *Scutellospora calospora* spores into 5 mL of 0.1% Tween 20 to obtain a final concentration of 100 spores per mL.

Endophyte-free and surface-sterilized seeds were separately treated with spore suspension by soaking them into the solution for 2 h under constant shaking at 60 rpm. Later, seeds were removed from spore suspension and dried under laminar air flow for 15 min. Commercially available MS (Murashige and Skoog, 1962) media were prepared (half strength) and autoclaved for 15 min at 121^0^C and 15 lbs pressure. After cooling, media were poured into petri plates. SHAM was filter sterilized and was added into MS media plates at final concentrations of 5mM/10mM. Three different sets of MS media plates were prepared: one set containing 5mM SHAM, one set containing 10mM SHAM and another without SHAM. 40 seeds were inoculated in each petri dish and two petri dishes were maintained for each treatment. After inoculation, plates were incubated under dark conditions until 40 h and shifted to light after 40 h. Germination rate and root length were calculated at two different time points, i.e., 40 and 65 h. Seeds germinated were observed in both petri dishes of each treatment and root length is calculated for seeds in both petri dishes. Germination rate was computed manually from each plate, and root length was measured using Trinocular Stereo zoom microscope (Magnus).

For germination percentage and root length, ANOVA with Tukey post-hoc test and Kruskal-Wallis test were performed for each time, respectively. Influence of factors (AMF inoculation and SHAM concentration) in germination is indicated in the corresponding graphs. Differences in root length between treatments for each time are stated with different letters (α = 0.05).

**Seed vigor characterization and prediction of field performance**

**Fig. D (i):** Control and primed, coated seeds of cv. Nerac 2 provided by Bejo Samen (Germany) were germinated in parallel at 25°C in the dark in petri dishes on 4 layers of commercial paper towel. 5mM and 10mM SHAM was prepared without adding DMSO by gradual dilution in heated bi-distilled water. Germination were recorded. Statistical significance of differences between control and primed seeds were tested according to the unpaired Student’s *t*-test.

**D (ii):** Organic carrot seeds (cultivar Nantaise 2/Milan) were provided by Bingenheimer Saatgut (Germany) in the frame of the EU LIVESEED project. Subsamples of 100 g seeds were equilibrated at 30% RH and subsequently experimentally aged by storing for 2, 3 or 5 weeks at 20 °C in steel tanks filled with 200 bar air providing an elevated partial pressure of oxygen (EPPO) of 42 bar (4.2 MPa) (Groot et al., 2012). At the end of the storage period the pressure was controlled released with a maximum pressure decline of 0.5% per minute. Seeds that had not been stored under pressure served as control. After storage the seed samples were vacuum packed in laminated aluminium foil pouches and stored in the freezer until further analysis. Germination tests were performed of blotters at 20 °C in the dark, with two replicates of 90 seeds per treatment. Seed with roots protruding at least 1 mm were scored as germinated. Scoring was done daily till ten days after sowing. Germination parameters as Maximum Germination, t_50_ and Area Under the Curve were calculated using Germinator software(Joosen et al., 2010). SHAM treatment was performed as described for Figure D (i). Statistical significance of differences between control and EPPO-treated seeds were tested according to the unpaired Student’s *t*-test.

**D (iii):**206 pea inbred lines were evaluated for dry-matter grain yield and total biological yield under rain-fed conditions in three autumn-sown Italian environments: Lodi (northern Italy; 45°19ʹN, 9°30ʹE) in cropping years 2013-14 and 2014-15, and Perugia (central Italy; 43°06ʹN, 12°23ʹE) in cropping year 2013-14. Each experiment was designed as randomized complete block with three replicates, with plots of 0.96 m^2^ size. Lines belonged to two recombinant inbred line (RIL) populations originated from crosses with cultivars ‘Isard’ with ‘Attika’ or ‘Kaspa’, which displayed high and stable grain yield in earlier multi-environment testing (Annicchiarico and Iannucci, 2008). Populations are designated hereafter as AI and KI from the initials of their respective parents. AI included 102 lines, and KI 104 lines. Results for Lodi and Perugia in 2013-14 were reported by Annicchiarico et al. (2019). Compared with these cropping environments, Lodi in 2014-15 was characterized by greater plant mortality (averaging 13 % over populations) due to colder winter (absolute minimum daily temperature = –11.6˚C).

Three lines per population, namely, one top-performing, one mid-ranking and one bottom-ranking for grain yield averaged across the three test environments, were selected from each RIL population for this study. Since aim was to assess the ability of calorespirometric prediction the intrinsic grain and/or biological yielding ability of lines, we imposed that mid-ranking and bottom-ranking lines within each RIL had to possess similar cold tolerance (as winter survival in coldest environment) and crop length as the top-yielding one (thereby avoiding any bias on results due to lack of key adaptive traits). Assessment across two genetic backgrounds (two RIL populations), and thorough phenotyping under agricultural conditions, were expected to ensure reliability of results obtained for calorespirometry.

**Calorespirometry for pea genotype characterization**: due to the larger size of seeds compared to carrot, single seed measurements were carried out. Seeds of the same cross (AI or KI) were germinated in parallel on filter paper in petri dishes (same size and same amount of added distilled water) at 25°C in the dark. At 10HAI, a single seed of each ranking level for yield ability of each cross (low, middle, high AI or KI) was placed into one of the three ampules and measured in parallel. 10 measurements according to 10 biological repetitions for each genotype were performed as such per cross. Oxycaloric equivalents (Rq/RCO2) were calculated as described (Arnholdt-Schmitt, 2017). Plot data of grain yield and biological yield were analysed by ANOVA including genotype, environment and block as fixed factors, comparing line mean values over environments by Newman-Keul’s test. Statistical analyses were performed using Statistical Analysis System (SAS) software.

**D (iv):** Oxycaloric equivalents (Rq/RCO_2_) of commercially available Desi and Kabuli chickpea seed types were measured individually in 12 biological repetitions. Statistical significance was tested by unpaired Student’s *t*-test.

**Expression analysis of *AOX* genes from the available transcriptomic data using RNA-seq data**

**Table 1 and Table S1:** *AOX* expression analysis was performed according to Saraiva et al. (2016). AOX expression data were obtained by using the transcriptomic studies (experimental results) available publically in Genbank (NCBI). NCBI Bioprojects provided PRJNA439301, PRJNA422335, PRJNA3089, PRJNA419307 were published only in SRA Genbank (NCBI), but not published by authors in any paper until this moment. In this method, the number of mapped reads on each gene was used to estimate the AOX gene expression, and then normalized using the RPKM (Reads Per Kilobase of transcript per Million of mapped reads). Gene expression data were statistically analyzed using the Prism tool (Graphpad Prism), through the variance analysis by ANOVA with the Tukey test parameters. Lower case letters represent the comparison of treatment conditions. The same letter indicates that no statistic difference between time/treatments was observed.

**References**

Annicchiarico, P., and Iannucci, A. (2008). Adaptation strategy, germplasm type and adaptive traits for field pea improvement in Italy based on variety responses across climatically contrasting environments. *Field Crops Res.,* 108, 133-142.

Annicchiarico, P., Russi, L., Romani, M., Pecetti, L., and Nazzicari, N. (2019). Farmer-participatory vs. conventional market-oriented breeding of inbred crops using phenotypic and genome-enabled approaches: a pea case study. *Field Crops Res*., 232, 30-39.

Arnholdt-Schmitt, B. (2017). “Respiration traits as novel markers for plant robustness under the threat of climate change: a protocol for validation”, in Plant Respiration and Internal Oxygen, ed. Gupta KJ (Humana Press, New York, NY), 183-191. ISBN: 978-1-4939-7292-0.

Basra, A. S., Sarlach, R. S., and Malik, C. P. (1989). Seed germination of *Panicum maximum* Jacq. in relation to respiratory inhibitors and gibberellic acid treatments. *Indian J. Plant Physiol.*, 32, 139-143.

Botha, F. C., Small, J. G. C., and Grobbelaar, N. (1986). The effect of cyanide, SHAM and azide on the germination and respiration of *Citrullus lanatus* seeds. *S Afr J Bot.,* 52(1), 77-80.

Campos, M. D., Nogales, A., Cardoso, H. G., Kumar, S. R., Nobre, T., Sathishkumar, R., and Arnholdt-Schmitt, B. (2016). Stress-induced accumulation of DcAOX1 and DcAOX2a transcripts coincides with critical time point for structural biomass prediction in carrot primary cultures (*Daucus carota* L). Front Genet., 7, 1.

Frederico, A. M., Campos, M. D., Cardoso, H. G., Imani, J., and Arnholdt‐Schmitt, B. (2009). Alternative oxidase involvement in *Daucus carota* somatic embryogenesis. Physiol. Plant., 137(4), 498-508.

Gamborg, O. L., Miller, R., and Ojima, K. (1968). Nutrient requirements of suspension cultures of soybean root cells. *Exp. Cell Res.,* 50(1), 151-158.

Groot, S. P. C., Surki, A. A., De Vos, R. C. H., and Kodde, J. (2012). Seed storage at elevated partial pressure of oxygen, a fast method for analysing seed ageing under dry conditions. *Ann. Bot.,* 110(6), 1149-1159.

Hansen, L. D., Criddle, R. S., and Smith, B. N. (2005). “Calorespirometry in plant biology”, in Plant Respiration: Advances in Photosynthesis and Respiration (vol. 18), eds. Lambers H., and Ribas-Carbo M. (Springer, Dordrecht), 17-30. ISSN: 978-1-4020-3589-0.

Joosen, R. V., Kodde, J., Willems, L. A., Ligterink, W., van der Plas, L. H., and Hilhorst, H. W. (2010). Germinator: A software package for high‐throughput scoring and curve fitting of Arabidopsis seed germination. *Plant J.,* 62(1), 148-159.

Murashige, T., and Skoog, F. (1962). A revised medium for rapid growth and bio assays with tobacco tissue cultures. *Physiol. Plant.,* 15(3), 473-497.

Velada, I., Cardoso, H. G., Ragonezi, C., Nogales, A., Ferreira, A., Valadas, V., and Arnholdt-Schmitt, B. (2016). Alternative oxidase gene family in *Hypericum perforatum* L.: characterization and expression at the post-germinative phase. Front. Plant Sci., 7, 1043.

**Supplementary Figures**


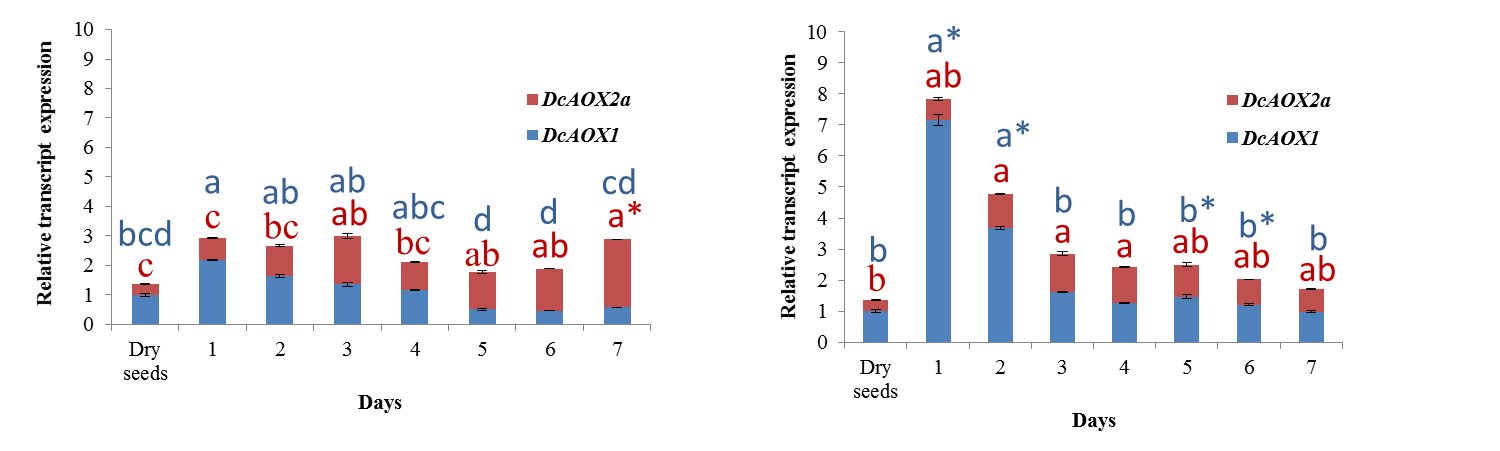


**(i) Auxin-free medium (ii) Auxin-containing medium**

**Figure S1. Relative transcript accumulation of *DcAOX1* and *DcAOX2* in carrot seeds cultured on auxin-free (initiation of germination) vs auxin-containing medium (SE-induction)**

*DcAOX1* and *DcAOX2a* relative transcript accumulation (mean ± SD) were analysed using *EF1α* and *β-Actin* as references. Two-way (treatment x time) ANOVA was applied followed by Tukey: differences between times inside a treatment are stated with different letters and differences between treatments inside a time with asterisks (α = 0.05). To keep ANOVA pre-requisites, variables were transformed: square root neperian logarithm for *AOX1* and square root for *AOX2a.*

**(i) Germination rate (%) (ii) Root length (µm)**

**Figure S2.** Influence of SHAM concentration and AMF on germination rate and emerging root length of surface-sterilized carrot seeds (mean ± SE).

**Table S1: Expression of *AOX* genes in available transcriptomic data from some angiosperms in response to endophyte interaction.**

| Experiment types | Species | Bioproject | Tissue/  Genotype | Sample | Repeat number | Gene expression (RPKM) | | | | | | | | | | | Reference |
| --- | --- | --- | --- | --- | --- | --- | --- | --- | --- | --- | --- | --- | --- | --- | --- | --- | --- |
|  |  |  |  |  |  | *AOX1a* | | *AOX1b* | | *AOX1c* | *AOX1d* | | *AOX1e* | *AOX2* | | Fungal endophyte *AOX* |  |
|  |  |  |  |  |  | *AOX1a1* | *AOX1a2* | *AOX1b1* | *AOX1b2* |  | *AOX1d1* | *AOX1d2* |  | *AOX2a* | *AOX2d* |  |  |
| Monocots plants endophytic infection (fungal or bactéria symbionts) | *Festuca arundinacea* | PRJNA284541 | Pseudostem | Non infected | 3 | 2.1^a^ | | Not  present | | 0.36 ^a^ | 0.75 ^a^ | | Not present | Not  present | | 0 ^a^ | Dinkins et al. 2017 |
|  |  |  |  | Infected with *Epichloe coenophila*4W | 3 | 1.65 ^a^ | |  |  | 0.26 ^a^ | 0.66 ^a^ | |  |  |  | 0.4 ^a^ |  |
|  |  |  | Leaf | Non infected | 3 | 1.4 ^a^ | |  |  | 22.8^b^ | 1.3 ^a^ | |  |  |  | 0 ^a^ |  |
|  |  |  |  | Infected with *E. coenophila*4W | 3 | 0.7^b^ | |  |  | 27 ^c^ | 0.84 ^a^ | |  |  |  | 0 ^a^ |  |
|  |  |  | Root | Non infected | 3 | 5.05^c^ | |  |  | 0.1 ^a^ | 7.77^b^ | |  |  |  | 0 ^a^ |  |
|  |  |  |  | Infected with *E. coenophila*  4W | 3 | 5.48^c^ | |  |  | 0.2 ^a^ | 4.66^c^ | |  |  |  | 0 ^a^ |  |
|  | *Lolium perenne* | PRJNA292034 | Sheat | Non infected | 2 | 8.1 ^a^ | | Not  present | | 6 ^a^ | 0.99 ^a^ | | Not present | Not  present | | 0 ^a^ | Schmid et al. 2017 |
|  |  |  |  | Infected with *E.festucae* | 2 | 6.8 ^a^ | |  |  | 10.4 ^a^ | 5.37^b^ | |  |  |  | 1.57^b^ |  |
|  |  |  | Blade | Non infected | 2 | 6.3 ^a^ | |  |  | 23.9^b^ | 1.39 ^a^ | |  |  |  | 0 ^a^ |  |
|  |  |  |  | Infected with *E.festucae* | 2 | 5.4 ^a^ | |  |  | 25.7^b^ | 16.5^c^ | |  |  |  | 0.32 ^a^ |  |
|  |  |  | Leaf | Non infected | - | 8.5 | |  |  | 20.1 | 0.9 | |  |  |  | 0 |  |
|  |  |  |  | Infected with *E.festucae* | - | 8.1 | |  |  | 21.1 | 1.48 | |  |  |  | 0.17 |  |
|  |  |  | Root | Non infected | - | 7 | |  |  | 0.06 | 3.07 | |  |  |  | 0 |  |
|  |  |  |  | Infected with *E.festucae* | - | 6.1 | |  |  | 0.06 | 11.4 | |  |  |  | 0 |  |
|  |  |  | Stem | Non infected | - | 3.9 | |  |  | 2.4 | 0.12 | |  |  |  | 0 |  |
|  |  |  |  | Infected with *E.festucae* | - | 4.3 | |  |  | 1.3 | 0.63 | |  |  |  | 0.23 |  |

| Monocots plants endophytic infection | *Achnatherum inebrians* | PRJEB12328 | Seeds | Non infected  2d | - | 1.5 | 0.2 | Not present | 0.17 | 0 | Not present | Not present | Not present | Chen et al. 2016 |
| --- | --- | --- | --- | --- | --- | --- | --- | --- | --- | --- | --- | --- | --- | --- |
|  |  |  |  | Infected with *Epichloegansuensis*  2d | - | 0.8 | 0.3 |  | 0.27 | 0 |  |  |  |  |
|  | *Oryza sativa* | PRJNA422335 | Shoot and roots | Control mock | 2 | 5.75 ^a^ | | Not  present | 12.14 ^a^ | 2.38 ^a^ | 0.56 ^a^ | Not  present | Not  present | Not published |
|  |  |  |  | Infected with endophyte | 2 | 5.9 ^a^ | |  | 7.2^b^ | 2.8 ^a^ | 0.65 ^a^ |  |  |  |
|  |  |  |  | Salt stress | 2 | 20.6^b^ | |  | 10.3 ^ab^ | 11.7^b^ | 0 ^a^ |  |  |  |
|  |  |  |  | Salt stress + endophyte | 2 | 25.4 ^b^ | |  | 8.2 ^b^ | 15.6 ^b^ | 0 ^a^ |  |  |  |
| Eudicots plants endophytic infection | *Arabidopsis thaliana* | PRJNA399521 | Seedlings 17d | Control mock | 3 | 12.28 ^a^ | | 0 ^a^ | 0.24 ^a^ | 0.07 ^a^ | Not  present | 0.04 ^a^ | Not  present | de Zélicourt et al. 2018 |
|  |  |  |  | Infected with *Enterobacter* sp. | 3 | 9.76^b^ | | 0 ^a^ | 0.14 ^a^ | 0.1 ^a^ |  | 0.01 ^a^ |  |  |
|  |  |  |  | Salt stress | 3 | 14^c^ | | 0 ^a^ | 0.2 ^a^ | 0.3 ^a^ |  | 0.03 ^a^ |  |  |
|  |  |  |  | Salt stress + *Enterobacter*sp. | 3 | 9.67^b^ | | 0 ^a^ | 0.08 ^a^ | 0.03 ^a^ |  | 0.03 ^a^ |  |  |

| Eudicots plants endophytic infection | *Lotus japonicus* | PRJNA3089 | Roots from wild type MG-20, 7d | Non infected | 3 | 18.7^a^ | Not  present | | Not  present | Not  present | Not  present | 5.8 ^a^ | 19.8 ^a^ | Not  present | Not published |
| --- | --- | --- | --- | --- | --- | --- | --- | --- | --- | --- | --- | --- | --- | --- | --- |
|  |  |  |  | Infected with *Mesorhizobium sp.* | 3 | 25^b^ |  |  |  |  |  | 7.9 ^a^ | 23.8 ^a^ |  |  |
|  |  |  | Roots from mutant vag-1, 7d (defficient in nodule construction) | Non infected | 3 | 13.38^c^ |  |  |  |  |  | 9.47^b^ | 37.6^b^ |  |  |
|  |  |  |  | Infected with *M. sp.* | 3 | 19.52 ^a^ |  |  |  |  |  | 10.3^b^ | 42.3^b^ |  |  |
|  |  | PRJNA384655 | Root – 15d | Non infected | 3 | 60.1 ^a^ | Not  present | | Not  present | Not  present | Not  present | 17 ^a^ | 26.46 ^a^ | Not  present | Kelly et al. 2018 |
|  |  |  |  | Infected with *M. sp.* | 3 | 47.1^b^ |  |  |  |  |  | 17.7 ^a^ | 27.1 ^a^ |  |  |
|  |  |  | Shoot – 15d | Non infected | 3 | 2.45^c^ |  |  |  |  |  | 27.7^b^ | 17.5^b^ |  |  |
|  |  |  |  | Infected with *M. sp.* | 3 | 2.41^c^ |  |  |  |  |  | 31.6^b^ | 10.9^b^ |  |  |
|  |  | PRJDB3234 | Root – 30d | Non infected | 3 | 12.34 ^a^ | Not  present | | Not  present | Not  present | Not  present | 11.21 ^a^ | 68.85 ^a^ | 0 ^a^ | Takeda et al. 2015 |
|  |  |  |  | Infected with *Rhizophagus irregulares* | 3 | 12.76 ^a^ |  |  |  |  |  | 15.05 ^a^ | 95.82^b^ | 2.42^b^ |  |
|  | *Helianthus annuus* | PRJNA417544 | Roots | Non infected – 4d | 3 | 0 ^a^ | 0.22 ^a^ | 2 ^a^ | Not  present | 1.45 ^a^ | Not  present | 42 ^a^ | | 0 ^a^ | Vangelist et al. 2018 |
|  |  |  |  | Infected with *R. irregulares*– 4d | 3 | 0.04 ^a^ | 0.27 ^a^ | 2.3 ^a^ |  | 0.52^b^ |  | 33.5 ^a^ | | 0.038 ^a^ |  |
|  |  |  |  | Non infected – 16d | 3 | 0 ^a^ | 37^b^ | 48^b^ |  | 1 ^a^ |  | 37.8 ^a^ | | 0 ^a^ |  |
|  |  |  |  | Infected with *R. irregulares*– 16d | 3 | 0 ^a^ | 30^b^ | 47.4^b^ |  | 0.82 ^a^ |  | 33.7 ^a^ | | 5.7^b^ |  |

| Eudicots plants endophytic infection | *Solanum lycopersicum* | PRJEB5335 | Seedlings | Non infected | 2 | 84.8 ^a^ | | Not present | | 0 ^a^ | 0.6 ^a^ | Not present | 3 ^a^ | 0 ^a^ | Zouari et al. 2014 |
| --- | --- | --- | --- | --- | --- | --- | --- | --- | --- | --- | --- | --- | --- | --- | --- |
|  |  |  |  | Infected with *Funneliformis mosseae* | 2 | 20^b^ | |  |  | 0 ^a^ | 0.14 ^a^ |  | 3.6 ^a^ | 0 ^a^ |  |
|  | *Papaver somniferum* | PRJNA419307 | Leaves | Non infected | 2 | 4.81 ^a^ | 12.64 ^a^ | 5.35 ^a^ | 16.05 ^a^ | Not  present | Not  present | Not  present | 13.65 ^a^ | Not  present | Not published |
|  |  |  |  | Infected with *Peronospora* sp. | 2 | 9.9^b^ | 39.6^b^ | 10.1^b^ | 50^b^ |  |  |  | 42^b^ |  |  |

**References**

Chen, N., He, R., Chai, Q., Li, C., and Nan, Z. (2016). Transcriptomic analyses giving insights into molecular regulation mechanisms involved in cold tolerance by Epichloë endophyte in seed germination of Achnatherum inebrians. Plant Growth Regul., 80:367–375.

de Zélicourt A., Synek L., Saad, M. M., Alzubaidy, H., Jalal, R., Xie, Y., Andrés-Barrao, C., Rolli, E., Guerard, F., Mariappan, K. G., Daur, I., Colcombet, J., Benhamed, M., Depaepe, T., Van Der Straeten, D., and Hirt, H. (2018). Ethylene induced plant stress tolerance by *Enterobacter* sp. SA187 is mediated by 2-keto-4-methylthiobutyric acid production. PLoS Genet. 14(3):e1007273.

Dinkins R. D., Nagabhyru P., Graham M. A., Boykin D., and Schardl C. L. (2017). Transcriptome response of Lolium arundinaceum to its fungal endophyte Epichloëco enophiala. New Phytol., 213 324–337.

Kelly, S., Mun, T., Stougaard, J., Ben, C., and Andersen, S. U. (2018). Distinct *Lotus japonicas* transcriptomic responses to a spectrum of bacteria ranging from symbiotic to pathogenic. *Front Plant Sci.,* 9:1218.

Saraiva, K.D., Oliveira, A.E., Santos, C.P., Lima, K.T., Sousa, J.M., Melo, D.F., and Costa, J.H. (2016). Phylogenetic analysis and differential expression of EF1α genes in soybean during development, stress and phytohormone treatments. Mol Genet Genomics., 291, 1505-1522.

Schmid, J., Day, R., Zhang, N., Dupont, P.Y., Cox, M.P., Schardl, C.L., Minards, N., Truglio, M., Moore, N., Harris, D.R., and Zhou, Y. (2017). Host tissue environment directs activities of an *Epichloë* endophyte, while it induces systemic hormone and defense responses in its native perennial ryegrass host. *Mol Plant Microbe Interact.,* 30(2):138-149.

Takeda, N., Handa, Y., Tsuzuki, S., Kojima, M., Sakakibara, H., and Kawaguchi, M. (2015). Gibberellins interfere with symbiosis signaling and gene expression and alter colonization by arbuscular mycorrhizal fungi in *Lotus japonicus. Plant Physiol.* 167(2):545-57.

Vangelisti, A., Natali, L., Bernardi, R., Sbrana, C., Turrini, A., Hassani-Pak, K., Hughes, D., Cavallini, A., Giovannetti, M., and Giordani, T. (2018) Transcriptome changes induced by arbuscular mycorrhizal fungi in sunflower (*Helianthus annuus* L.) roots. *Sci Rep.* 8(1):4.

Zouari, I., Salvioli, A., Chialva, M., Novero, M., Miozzi, L., Tenore, G.C., Bagnaresi, P., and Bonfante, P. (2014) From root to fruit: RNA-seq analysis shows that arbuscular mycorrhizal symbiosis may affect tomato fruit metabolism. *BMC Genomics.,* 15:221.
